# Single Bout Exercise in Children with Juvenile Idiopathic Arthritis: Impact on Inflammatory Markers

**DOI:** 10.1155/2018/9365745

**Published:** 2018-06-13

**Authors:** Rochette Emmanuelle, Duché Pascale, Hourdé Christophe, Evrard Bertrand, Pereira Bruno, Echaubard Stéphane, Merlin Etienne

**Affiliations:** ^1^Pédiatrie Générale Multidisciplinaire, Hôpital Estaing, CHU Clermont-Ferrand, 63000 Clermont-Ferrand, France; ^2^Université Clermont Auvergne, INSERM, CIC 1405, Unité CRECHE, 63000 Clermont-Ferrand, France; ^3^Laboratoire des Adaptations Métaboliques en conditions Physiologiques et Physiopathologiques (AME2P), Université Clermont Auvergne, EA 3533, Clermont-Ferrand, France; ^4^CRNH-Auvergne, 63000 Clermont-Ferrand, France; ^5^Laboratoire Interuniversitaire de Biologie de la Motricité (E A7424), Université Savoie Mont Blanc, 73376 Le Bourget-du-Lac, France; ^6^Université Clermont Auvergne, INRA, UMR 1019 UNH, ECREIN, 63000 Clermont-Ferrand, France; ^7^Service d'Immunologie, CHU Clermont-Ferrand, 63000 Clermont-Ferrand, France; ^8^CHU Clermont-Ferrand, Délégation de la Recherche Clinique et Innovations, 63000 Clermont-Ferrand, France

## Abstract

**Objective:**

In a context of inflammatory disease such as juvenile idiopathic arthritis (JIA), we do not know what impact physical activity may have on a deregulated immune system. The objective is to measure the impact of a single bout of exercise on plasma inflammatory markers such as calprotectin, IL-6, sIL-6R, sgp130, and the hypothalamic-pituitary-adrenal axis in children with juvenile idiopathic arthritis.

**Methods:**

Twelve children with JIA performed a nonexercise control day and a consecutive day that included a 20 min exercise bout at 70% of max-HR at 08:30 am. Venous blood samples were taken at 08:30, 08:50, 09:30, 10:30 am, and 12:00 pm to measure plasma concentrations of calprotectin, IL-6, sIL-6R, sgp130, cortisol, and ACTH. Pain was evaluated at 08:30, 08:50 am, and 06:00 pm.

**Results:**

There was a transient twofold increase in postexercise self-evaluated pain (*p* = 0.03) that disappeared in the evening. A single bout of exercise resulted in a 1.7-fold increase in plasma calprotectin (*p* < 0.001) but not IL-6 and its soluble receptors. Calprotectin levels returned to baseline within 3 hours after cessation of exercise.

**Conclusion:**

Acute exercise in children with JIA induced slightly musculoskeletal leg pain and transient increased plasma calprotectin levels but not IL-6 levels. Trial registration in ClinicalTrials.gov, reference number NCT 02502539, registered on 29 May 2015.

## 1. Introduction

Juvenile idiopathic arthritis (JIA), the most common inflammatory pathology in children, is a chronic disease characterized by persistent joint inflammation due to immune system disruption [1]. Some of the proteins that perpetuate inflammatory mechanisms in JIA are calgranulins (myeloid-related protein (MRP)-8 and MRP-14). They are secreted by activated monocytes and phagocytes and induce the expression of proinflammatory cytokines (IL-1*β*, IL-6, or TNF) [2]. In JIA, the MRP8/14 complex, also called calprotectin, is a reliable marker of subclinical disease activity [3, 4].

It has emerged that exercise may have direct effects not only on metabolic and fitness parameters but also on the pathogenesis of autoimmune diseases as it attenuates chronic low-grade systemic inflammation [5]. After acute exercise of varying intensities and lengths, plasma calprotectin levels in healthy adults increase at a rate of between 3.4-fold (VO_2_ max test) and 96.3-fold (marathon) [6]. Although IL-6 is known as a potent proinflammatory signal (involved in the pathology of JIA), the transient rise in circulating IL-6 during exercise appears to have anti-inflammatory effects [7]. In addition, IL-6 released from contracting muscle acts in a hormone-like manner to mobilize extracellular substrates or inhibits low-level TNF-*α* production. Likewise, physical activity is known to impact the hypothalamic-pituitary-adrenal (HPA) axis in healthy children [8-10] and modulate the anti-inflammatory effect of cortisol. However, the disturbed HPA axis in JIA leads to impaired secretion of adrenocorticotropic hormone (ACTH) and cortisol [11, 12]. Even though physical activity is increasingly recognized as a useful intervention in patients with rheumatisms [13], to our knowledge, there is no data on the effect of acute bouts of physical activity on IL-6 and its receptors, calprotectin and HPA axis in children with inflammatory disease.

Here, we set out to explore the impact of an acute bout exercise on pain and on the secretion of molecules involved in the inflammatory process in JIA.

## 2. Methods

Twelve children aged 8 to 16 years with nonsystemic JIA according to the criteria of the International League of Associations for Rheumatology were enrolled in the study and were followed at the pediatric unit of Clermont-Ferrand University Hospital in France.

Patients were excluded if they had a physician-diagnosed infection, had been treated by IL-6 blockade in the last 6 months, and had received oral corticosteroids within the last 3 months. Treatments were continued during the study.

The study was approved by the governing ethics committee (Comité de Protection des Personnes Sud-Est VI, reference number: AU1190). All participants and their parents have given consent.

Experimental design consisted of two consecutive days (day 1: control, day 2: exercise day).

Pain was self-evaluated by the patient on a visual analog scale at 08:30, 08:50 am, and 06:00 pm during the 2 days.

The exercise test consisted of a 20 min bout, between 08:30 am and 08:50 am, at 70% of the maximal theoretical heart rate (max-HR) performed on a cycle ergometer (Ergo-metrics 800S, Ergoline, Bitz, Germany) during day 2. Theoretical max-HR was defined as 220-age.

Venous blood samples were taken via an indwelling catheter at 08:30, 08:50, 09:30, 10:30 am, and 12:00 pm, into EDTA K2 Vacutainer® tubes (Becton Dickinson, Franklin Lakes, NJ, USA) for analysis of ACTH, IL-6, sIL-6R, sgp130, and calprotectin, into Vacutainer Lithium Heparin Tubes (Becton Dickinson, Franklin Lakes, NJ, USA) for analysis of hs-CRP and into Vacutainer SST II Advance Tubes (Becton Dickinson, Franklin Lakes, NJ, USA) for analysis of cortisol. Samples collected for calprotectin, IL-6, sIL-6R, and sgp130 were immediately centrifuged at 3000*g* for 10 min at 4°C and stored at −80°C until analysis, while all other blood samples were analyzed immediately.

Plasma ACTH levels were measured by chemiluminescent immunometric assay (Immulite 2000, Siemens Healthcare Diagnostics; analytical sensitivity = 5 pg/mL). Serum cortisol levels were measured by chemiluminescence on an ADVIA Centaur XP Analyzer (Siemens Healthcare Diagnostics; analytical sensitivity = 27.6 nmol/L). Plasma MRP8/14 levels were measured using commercially available ELISA kits (Bühlmann Laboratories, Schönenbuch, Switzerland; analytical sensitivity = 400 ng/mL). Plasma highly sensitive C-reactive protein (hs-CRP) levels were measured by nephelometry on a Dimension Vista® analyzer (Siemens Healthcare Diagnostics; analytical sensitivity = 0.16 mg/L). Plasma IL-6 levels were measured using commercially available ELISA kits (EH2IL65, Thermo Fisher Scientific, USA; analytical sensitivity < 1 pg/mL). Plasma sgp130 levels were measured using commercially available ELISA kits (EHIL6ST, Thermo Fisher Scientific, USA; analytical sensitivity = 4 pg/mL). Plasma sIL6-R levels were measured using commercially available ELISA kits (KHR0061, Thermo Fisher Scientific, USA; analytical sensitivity = <8 pg/mL). All analyses were made on duplicates.

Statistical analyses were performed using Stata software version 13 (StataCorp, College Station, TX, USA). Tests were two-sided, with a type I error set at *α* = 0.05. Continuous data were expressed as mean ± standard deviation (SD) or median (interquartile range) according to statistical distribution (assumption of normality assessed by using the Shapiro-Wilk test). To take into account between- and within-patient variability due to several measures for the same subject, random effects models for correlated data were performed rather than the usual statistical tests which would be inappropriate due to unverified assumption of independence. Time-point evaluations, sessions, and their interactions were considered fixed effects whereas a patient was a random effect (slope and intercept). The normality of residuals from these models was studied as described above. When appropriate, the data were log-transformed to achieve normality of the dependent endpoint.

## 3. Results

Baseline patient characteristics are reported in Table 1. Mean age was 12.3 ± 2.8 years, BMI was 19.7 ± 2.9 kg/m^2^, and disease duration was 29.4 ± 18.4 months. Mean fasting hs-CRP was 0.33 ± 0.4 mg/L.

**Figure 1:**
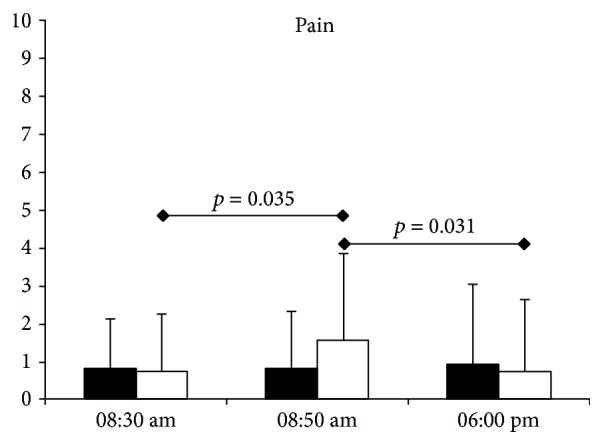
Pain evaluations by visual analog scale during the control day (black bar) and exercise day (white bar). Data are means ± 95% CI.

Exercise bout induced a transient but significant increase in pain postexercise that resolved in few hours (Figure 1). This musculoskeletal pain involved muscles and joints, mainly in the legs.

**Figure 2:**
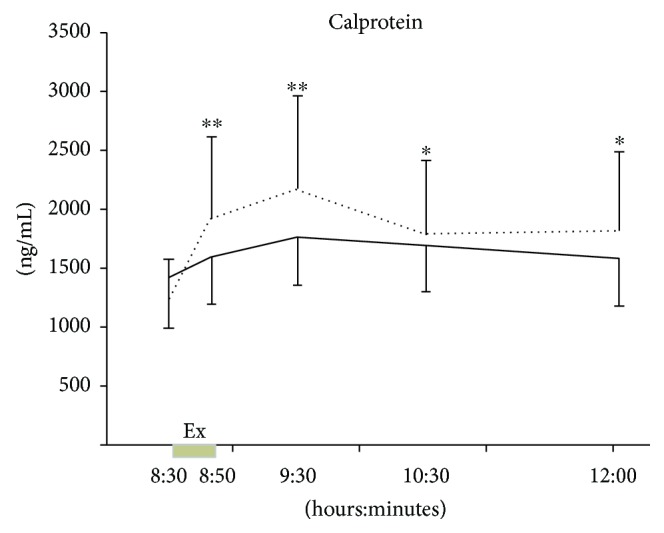
Plasma levels of calprotectin during the control day (solid line) and in response to 20 min of exercise (Ex) between 08:30 and 08:50 am (dotted line). Data are means ± 95% CI. ^∗^ Significantly different to baseline at *p* < 0.05. ^∗∗^ Significantly different to baseline at *p* < 0.001.

**Figure 3:**
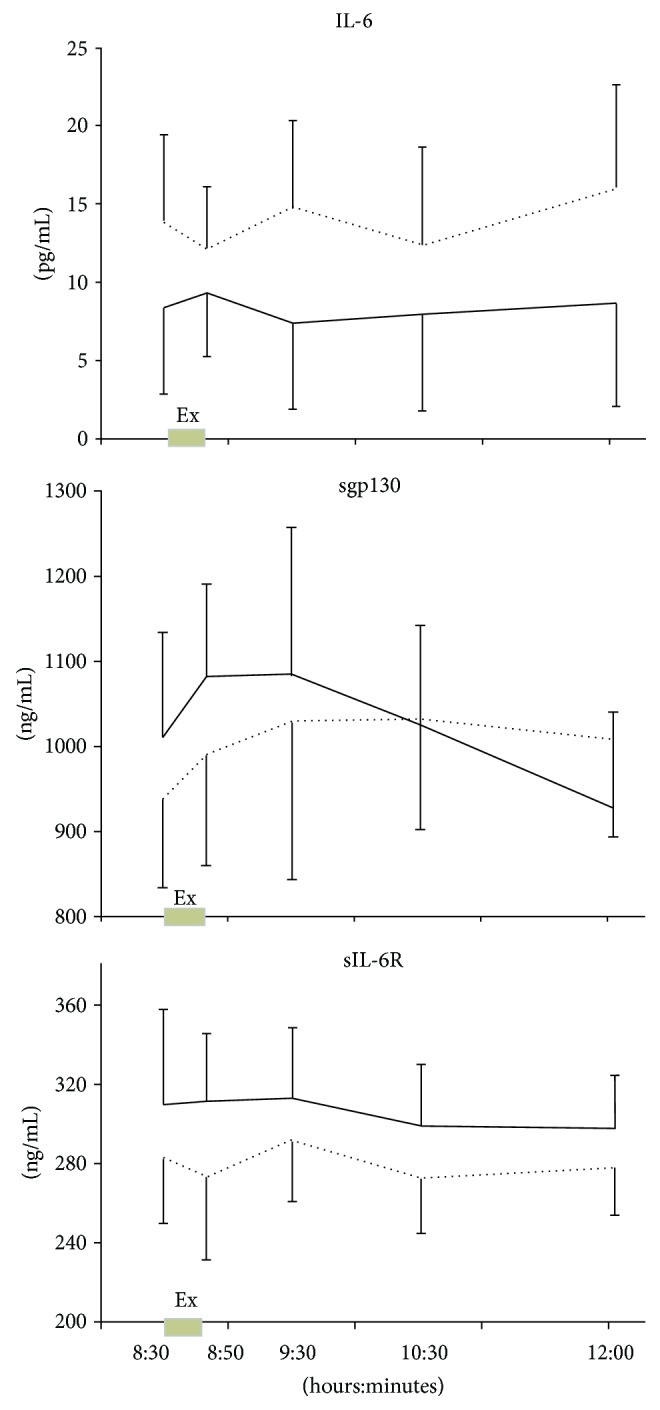
Plasma level of IL-6, sgp130, and sIL-6R during the control day (solid line) and in response to 20 min of exercise (Ex) between 08:30 and 08:50 am (dotted line). Area under the curve (AUC) for plasma sIL-6R decreased 8.4% between control day and exercise day (from 63.36 10^6^ ± 1.06 10^6^ to 58.03 10^6^ ± 0.97 10^6^; *p* = 0.006). AUC for plasma sgp130 increased 2.6% between control day and exercise day (from 188.96 10^6^ ± 4.81 10^6^ to 193.91 10^6^ ± 5.08 10^6^; *p* = 0.11). Data are means ± 95% CI.

Exercise induced a 1.7-fold increase in calprotectin level immediately after the exercise (*p* < 0.001) that returned to normal within three hours after the end of the exercise bout (Figure 2). However, there was no effect of exercise on IL-6, sIL-6R, and sgp130 levels (Figure 3).

**Figure 4:**
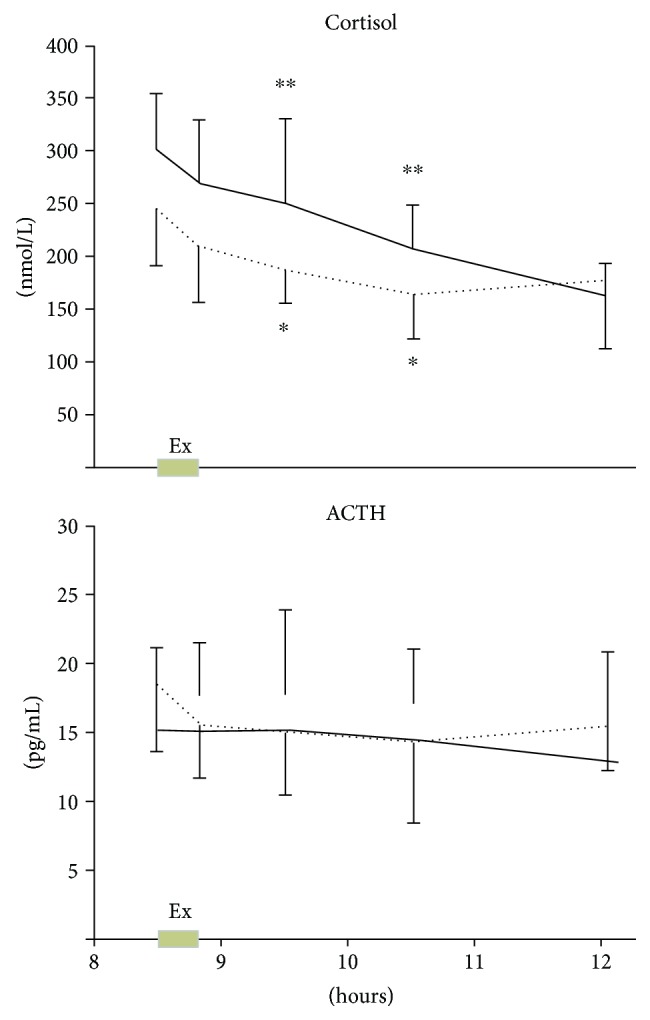
HPA axis secretion in children with JIA. Serum cortisol level and plasma ACTH level during the control day (solid line) and in response to 20 min of exercise (Ex) between 08:30 and 08:50 am (dotted line). ^∗^ Significantly different to baseline at *p* < 0.05. ^∗∗^ Significantly different to baseline at *p* < 0.001. Data are means ± 95% CI.

Area under the curve (AUC) for plasmatic ACTH and serum cortisol decreased, respectively, from 11.8% (from 3228 ± 128 to 2848 ± 124; *p* = 0.58) to 17.4% (from 43,455 ± 1294 to 35,943 ± 1217; *p* = 0.06) between the nonexercise control day and the exercise day (Figure 4).

## 4. Discussion

Overall, the exercise session was well tolerated in all children. There was a slight increase in pain immediately postexercise, but the effect was transient and disappeared before the evening. In terms of pain, it is therefore conceivable to advocate exercise in this child population, as more and more studies are showing that physical activity improves quality of life and symptoms in JIA patients [14, 15].

In healthy adults, acute exercise is known to induce a significant secretion of calprotectin [6] and IL-6 [7] by muscle in an exercise intensity-dependent manner. In fact, exercise promotes muscle recruitment of activated neutrophils, this phenomenon is followed by a systemic counterregulation involving IL-10 and IL-1ra [7]. Here, we observed a slight increase of calprotectin and no effect on IL-6. The absence of IL-6 increase could be explained by the short duration and a low intensity of the exercise bout tested. Furthermore, as exercise does not increase calprotectin levels in neutrophils [6], the likely source is skeletal muscle, since S100A8 and S100A9 mRNA rates increase in skeletal muscle during exercise [16]. Therefore, as our child population has a lower muscle mass and incomplete muscular recruitment [17], there is likely a lower production of calprotectin and IL-6 by the muscles than in adults. In addition, pubertal status, physical exercise training, and BMI seem to be factors involved in the impact of exercise-induced IL-6 release in children [18, 19]. Nevertheless, in our child population with systemic low-grade inflammation, as in studies in children with obesity or type 1 diabetes, single bout exercise is not followed by increased IL-6 levels [19]. Finally, the fact that our patients are under medication (methotrexate or TNF-*α* inhibitor) may also explain the discrepancy between the results found here and data in adult or healthy subjects. A calprotectin outbreak may have been feared in response to exercise in a context of proinflammatory disease, this is not the case in response to an acute exercise of moderate intensity.

IL-6 is involved not only in the activation of the immune system but also in regenerative processes and in the regulation of metabolism, the maintenance of bone homeostasis, and many neural functions [7, 20]. IL-6 is usually concerned as a proinflammatory cytokine. However, in the setting of muscular release, it is known to induce anti-inflammatory effects with inhibition of TNF-*α* production and may thus prevent TNF-*α*-induced insulin resistance [7]. Taken together, these observations raise questions over the different role for IL-6 in children compared to adults, making it necessary to address whether acute physical activity is followed by systemic counterregulation in children with JIA.

IL-6 acts through a heterodimeric signaling complex consisting of the IL-6 receptor (IL-6R) and the signal-transducing subunit glycoprotein 130 (gp130). Trans-signaling of IL-6 through its soluble receptors is involved in the promotion of chronic inflammatory diseases [21, 22]. Concerning the soluble receptors of IL-6, our results showed no effect of exercise on plasma sgp130 and sIL-6R levels. These results can be explained by the small number of patients and the fact that within-subject variation was lower than between-subject variation for IL-6, sIL-6R, and sgp130 in response to exercise, as reported in another study [23]. This finding can also in part explain why data on the effect of exercise on the rate of sgp130 is contradictory, with reports citing either an increase or a decrease or no effect [23-28]. Furthermore, all IL-6 family cytokines use gp130 as a receptor [29], and this may partly explain the high concentrations of this receptor and its homeostasis. Finally, our levels of sIL-6R and sgp130 are higher than reported in literature, although sIL-6R levels are high in the serum and synovial fluid of JIA patients [30]; this discrepancy could be explained by the fact that we have used plasma or by the detection kits used.

We did not observe any impact of physical activity on the HPA axis except for cortisol. The decrease in cortisol level observed on the second day was probably due to lower stress at this session, as the children were aware of the study environment, contrary to the first session.

The relatively small number of patients could be considered as a limitation. A second limitation is that it focused on the physiological response to exercise at blood level, which may not be an accurate reflection of the muscular or articular response in this population. However, muscle biopsies or articular aspirations in these children would have been complicated to perform. Nevertheless, further research is warranted to confirm our results and evaluate other markers involved in the inflammatory response to exercise in children (healthy or with an inflammatory pathology).

## 5. Conclusion

This study found that in children with JIA, exercise is followed by a transient mild proinflammatory systemic response and low musculoskeletal leg pain, which resolve within three hours.

## Figures and Tables

**Table 1:** Baseline demographics, clinical characteristics, and baseline laboratory findings.

Age (years)	Sex	BMI (kg/m^2^)	*Z* score	JIA subtype	Disease duration (months)	Concomitant DMARDs	hs-CRP (mg/L)^ǂ^	Heart rate during exercise: min–max (bpm)
11	F	14.8	−1.4	Psoriatic	16.8	MTX	0.24	141–149
10	M	18.1	+0.6	ERA	14.1	Sulfasalazine	<0.16	144–150
8	F	21.7	+1.8	pJIA RF−	4.9	MTX	1.4	142–152
9	M	15.5	−0.4	ERA	35.6	MTX	<0.16	143–152
14	F	19.7	+0.2	pJIA RF−	9.0	MTX	<0.16	140–149
14	M	20.1	+0.3	Psoriatic	40.1	Infliximab + leflunomide	0.33	141–149
16	F	21.1	+0.2	ERA	25.8	NSAIDs	0.53	138–147
9	M	21.3	+1.7	oJIA	55.2	NSAIDs	<0.16	140–151
15	F	23.1	+0.9	ERA	20.7	NSAIDs	<0.16	140–149
13	F	19.4	+0.2	pJIA RF+	23.0	MTX + etanercept	<0.16	138–150
12	F	16.9	−0.5	Undif	67	None	<0.16	139–151
16	F	24.4	+1.0	Undif	20.5	Etanercept	0.22	138–147

JIA: juvenile idiopathic arthritis; oJIA: oligoarticular JIA; pJIA RF−: rheumatoid factor-negative (RF−) polyarticular JIA; pJIA RF+: rheumatoid factor-positive (RF+) polyarticular JIA; ERA: enthesitis-related arthritis; Psoriatic: psoriatic JIA; Undif: undifferentiated; DMARDs: disease-modifying antirheumatic drugs; MTX: methotrexate; NSAIDs: nonsteroidal anti-inflammatory drugs; hs-CRP: high-sensitivity C-reactive protein; bpm: beats per minute. ^ǂ^Normal value < 3.0 mg/L. Values of <0.16 are below the minimum detectable limit of the kit.

## Data Availability

The data used to support the findings of this study are available from the corresponding author upon request.
