# Corrigendum to “Single Bout Exercise in Children with Juvenile Idiopathic Arthritis: Impact on Inflammatory Markers”

**DOI:** 10.1155/2018/2074269

**Published:** 2018-09-06

**Authors:** Emmanuelle Rochette, Pascale Duché, Christophe Hourdé, Bertrand Evrard, Bruno Pereira, Stéphane Echaubard, Etienne Merlin

**Affiliations:** ^1^Pédiatrie Générale Multidisciplinaire, Hôpital Estaing, CHU Clermont-Ferrand, 63000 Clermont-Ferrand, France; ^2^Université Clermont Auvergne, INSERM, CIC 1405, Unité CRECHE, 63000 Clermont-Ferrand, France; ^3^Laboratoire des Adaptations Métaboliques en conditions Physiologiques et Physiopathologiques (AME2P), Université Clermont Auvergne, EA 3533, Clermont-Ferrand, France; ^4^CRNH-Auvergne, 63000 Clermont-Ferrand, France; ^5^Laboratoire Interuniversitaire de Biologie de la Motricité (E A7424), Université Savoie Mont Blanc, 73376 Le Bourget-du-Lac, France; ^6^Université Clermont Auvergne, INRA, UMR 1019 UNH, ECREIN, 63000 Clermont-Ferrand, France; ^7^Service d'Immunologie, CHU Clermont-Ferrand, 63000 Clermont-Ferrand, France; ^8^CHU Clermont-Ferrand, Délégation de la Recherche Clinique et Innovations, 63000 Clermont-Ferrand, France

In the article titled “Single Bout Exercise in Children with Juvenile Idiopathic Arthritis: Impact on Inflammatory Markers” [1], the first and last names of all the authors were reversed. The revised authors' list is shown above.

## References

[1] Emmanuelle R., Pascale D., Christophe H. (2018). Single bout exercise in children with juvenile idiopathic arthritis: impact on inflammatory markers. *Mediators of Inflammation*.

